# Advances in biocultural geography of olive tree (*Olea europaea* L.) landscapes by merging biological and historical assays

**DOI:** 10.1038/s41598-020-64063-8

**Published:** 2020-05-06

**Authors:** Giuseppe Russo, Isacco Beritognolo, Marina Bufacchi, Vitale Stanzione, Andrea Pisanelli, Marco Ciolfi, Marco Lauteri, Stephen B. Brush

**Affiliations:** 1 Institute of Research on Terrestrial Ecosystems (IRET), National Research Council of Italy (CNR), Viale Marconi 2, 05010 Porano (TR), Italy; 2 Institute for Agricultural and Forest Systems in the Mediterranean (ISAFoM), National Research Council of Italy (CNR), Via della Madonna Alta 126, 06128 Perugia, Italy; 3 Department of Human Ecology, University of California, Davis, One Shields Avenue, Davis, CA 95616 USA

**Keywords:** Stable isotope analysis, Sustainability

## Abstract

Olive tree is a vector of cultural heritage in Mediterranean. This study explored the biocultural geography of extra virgin olive oil (EVOO) from the cultivar *Ogliarola campana* in Campania region, Italy. Here, the rich cultural elements related to olive tree and oil represent a suitable case study for a biocultural analysis. We joined analytical techniques, based on stable isotopes and trace elements of EVOOs, with humanistic analyses, based on toponymy and historical data. In order to provide a science-based assessment of the *terroir* concept, we set up a new method of data analysis that inputs heterogeneous data from analytical and anthropic variables and outputs an original global evaluation score, named *terroir score*, as a measure of biocultural distinctiveness of the production areas. The analysis highlighted two distinct cultural sub-regions in the production area of *Ogliarola campana*: a continental cluster in the inner area of Irpinia and a coastal one around Salerno province. Finally, a biocultural map displays the diversity of heterogeneous variables and may support science-based decision making for territory valorisation. This novel biocultural analysis is a promising approach to substantiate the *terroir* concept with science-based elements and appears suitable to characterize local agri-food products with old tradition and historical data.

## Introduction

The Mediterranean olive landscape integrates biological and cultural elements dating back several millennia^1^. Both identity persistence and evolution of such landscape are, at present, testified by the vitality of the value chain of extra-virgin olive oil (EVOO) at local, national and international level. This is accompanied by an increasing demand for labelling and regulatory systems, in order to designate the commodity’s geographic origin and to warrant its value^2^. In fact, the identity valorisation and protection of EVOO are tightly tied to the scientific assessment of its origin, as a way to solve the ambiguity of the geographic indication contained in the labels^3^. Thus, the EVOO characterization would reflect its own *terroir* system, based on its microclimate, soil and geomorphology^4^. The Italian consortium for olive cultivation (UNAPROL) recommends that “*the specialization of varieties and the typicality of the production connected to cultivars of high quality*” should be included in the Protected Designation of Origin (PDO) of EVOOs^5^. In this way, the Italian PDO regulation anticipated the recognition of *terroir*-like differences, but a standardized procedure to characterize EVOO *terroir* is missing. For this purpose, the pattern of variability in the characteristics of EVOOs at a small geographic scale may reveal new discriminating elements.

Genetic, sensory, and analytical tools have been set up to characterize and distinguish EVOOs^6,7^. DNA markers can be used to identify the olive cultivar of EVOOs, but their capacity to trace local productions may be limited because some cultivars are spread in many regions and several PDO labelled EVOOs include similar cultivar blends. The distinctiveness of EVOO production areas is most likely to be derived not only from cultivar identity, but rather from local agro-ecological conditions and *terroir*^4,8^. Analytical methods, based on the determination of stable isotopes of light elements, have been applied to trace the geographic origin of olive oils^9,10^. The ^13^C/^12^C and ^18^O/^16^O isotopic ratios in EVOOs and plant material have been applied as bioclimatic markers of the cultivation site and plant ecophysiological response^9–13^. A further approach to discriminate EVOOs’ geographic origin is the analysis of trace elements that can be taken up from soil to plants^14^ and the presence of heavy metals, which can also help in tracing specific cultivation areas^15^. Despite the power of the quoted technologies, the analytical characterization of EVOOs is insufficient for a complete comprehension of the EVOO landscape, which is the result of complex interactions between nature, society, and productive transformations in the course of history^16,17^. In this sense, the landscape contains values of intangible, cultural and aesthetic nature, which refer to the socio-economic, historical and cultural features of a territory^18,19^. The concept of *terroir* was originally developed for wine, but now encompasses many other crops, including olives. It refers to “delimited areas with homogeneous environmental features that confer typical wine qualities identified through collective memory and conveyed from generation to generation within a territory marked by social context and cultural technical choices”^20,21^. Thus, a *terroir* is shaped by a long heuristic process (lasting hundreds of years), which involves environment, as well as history, cultivation practices and market dynamics, as testified by grapevine selection inherited from medieval times and monasteries^21^. Thus a novel concept of *terroir* could include the anthropic and cultural elements.

**Figure 1:**
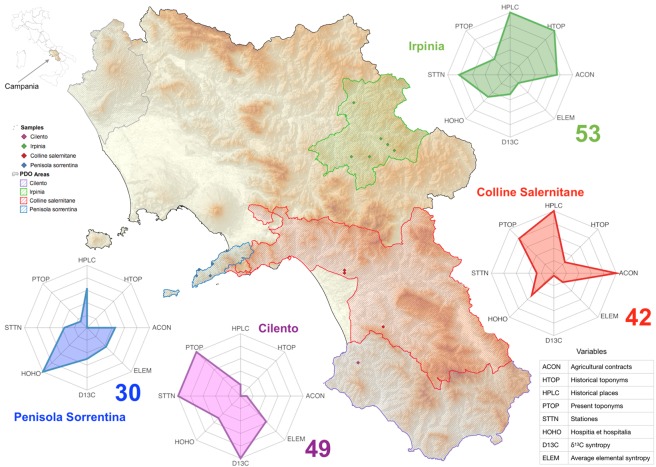
Map of Protected Designation of Origin (PDO) areas that produce the olive cultivar *Ogliarola campana* in Campania region, identified by a colour filling and border (colour code on the left). The sites of sampling are represented by diamond symbols on the map. For each PDO area, the radar plots display the *terroir score* (bold numeral) and its partitioning in the physical and anthropic variables (table bottom right). The map was generated with the software packages R^54^ (https://www.R-project.org), DataGraph from Visualtools (https://www.visualdatatools.com/DataGraph/), and QGIS^52^ (http://qgis.osgeo.org).

Our study addresses this novel concept of *terroir* in EVOO production areas of the Italian region Campania. This region, owing to its geographic and historical complexity, represents an ideal study area to develop a novel interpretation of the EVOO cultural landscape, which considers the oil analytical features and the cultural and historical elements of the territory^17^. We focused the study on the mono-varietal EVOOs obtained from the olive cultivar *Ogliarola campana*, which provides about 35% of the regional production of olives and is well-represented in four out of five EVOO PDOs of Campania region: Irpinia-Colline dell’Ufita, Penisola Sorrentina, Colline Salernitane and Cilento (Fig. 1)^22^. Campania region has a structured EVOO value chain and the historical importance of olive oil production is well documented since the Roman Age, as reported by Pliny the Helder in the *Naturalis Historia*. Later, the Christian monasticism drove the transformation of uncultivated lands, reprising the olive cultivation after the Early Middle Ages^23^. In the medieval Southern Italy, the strong influence of monasticism on the agriculture sector is testified by ancient sources and toponymy. Campania region is rich of toponyms related to agriculture, which form a complex “semantic network” revealed by their etymology^24^. The local toponyms with archaic origin reveal a background related to the late Latin or to the vernacular of the Early Middle Ages^25,26^, when the monasticism promoted the development of olive oil production in Campania. This rich geographic and historical information could aid in dissecting and interpreting the present biocultural diversity of EVOO landscape. The concept of biocultural diversity bases on the assumption of an inextricable link between biological (environment, fauna and flora) and cultural (history, religion, ethnics and language) diversity^27^. The UNESCO Florence Declaration^28^ recognizes the social importance of the biocultural diversity and recommends actions at regional level to implement certification and product labelling, and promote the competitiveness of local productions and rural societies. However, due to their limited extension, the local olive production systems cannot be interpreted according to the standard concept of biocultural diversity, which includes variables of humanity richness (e.g., languages, ethnicity, religion, technology) at a large geographic scale^29^. On the contrary, the toponymy can characterize production areas at a small scale, thus revealing the diversity of the local intangible heritage in terms of ancient practices of land use. The bio-environmental and cultural complexity of Campania region appears adequate to overcome the intrinsic limitations of biocultural studies at a small geographical scale^15^ and to adopt an analytical-classificatory-semantic study of toponyms^30^. The novelty of the present study is the utilization of both biological and cultural variables, in order to perform a cross-disciplinary analysis on the geographic framework. The study aims to: 1) characterize the EVOO production landscape by defining its biocultural diversity through a combination of chemical (stable isotopes and trace elements) and cultural-historical analyses (ancient documents and toponyms); 2) develop an original score evaluation methodology and a new index to measure the biocultural diversity of small EVOO production areas; 3) substantiate the *terroir* value and distinctiveness of EVOO areas in Campania region.

## Results

### Environmental features of production areas and chemical analysis of olive oil

The geographic and bioclimatic features were significantly heterogeneous across the four *Ogliarola* production areas under study (Table 1a). In particular, Irpinia (IR) was significantly separated from the other areas by most of the environmental variables. Among the oil chemical features, only δ^13^C and Ba were significantly different between the four areas (Table 1b).

**Table 1:** ^(a)^Geographical, meteorological and bioclimatic features of four production areas of the olive cultivar *Ogliarola campana* in Campania region. ^(b)^Stable carbon isotope composition (δ^13^C) and concentrations (μg*kg^-1^) of chemical elements in olive oils sampled in four production areas of Campania region.

Variable	ANOVA *p-value*	Kr.-Wal. *p-value*	Productions areas							
			Cilento		Colline Salernitane		Irpinia		Penisola Sorrentina	
			mean	(S.D.)	mean	(S.D.)	mean	(S.D.)	mean	(S.D.)
**a) Site environmental features**										
Latitude °	4.75*10^-10^		40.32^c^	(0.00)	40.59^b^	(0.08)	41.06^a^	(0.06)	40.63^b^	(0.02)
Sea dist. Km		0.0014	6.47	(0.00)	11.22	(2.16)	54.16	(6.15)	0.64	(0.59)
Elevation a.s.l.	6.9*10^-07^		348.00^b^	(0.00)	146.80^c^	(76.71)	652.86^a^	(128.21	120.25^c^	(63.88)
Av. Temp. °C	0.0001		15.08^ab^	(0.00)	14.21^b^	(0.92)	12.82^c^	(0.90)	16.09^a^	(0.53)
Rain fall, mm	0.0228		802.00^a^	(0.00)	686.26^ab^	(139.07)	619.43^b^	(28.23)	759.25^a^	(28.65)
Xeroth. Index	0.0007		57.20^ab^	(0.00)	80.18^a^	(25.78)	36.60^b^	(4.87)	62.50^a^	(4.39)
**b) EVOO chemical features**										
δ^13^C	0.0282		-29.13^b^	(0.18)	-29.79^ab^	(0.45)	-30.11^b^	(0.35)	-29.15^a^	(0.80)
Al		0.2267 ns	5.16	(2.68)	9.60	(9.67)	2.68	(0.61)	5.28	(1.77)
Ba	0.0190		0.20^b^	(0.16)	0.41^ab^	(0.30)	0.19^b^	(0.16)	0.68^a^	(0.23)
Ca	0.1410 ns		46.71	(27.31)	99.87	(74.90)	56.43	(48.14)	143.34	(67.64)
Cu	0.1380 ns		0.98	(1.38)	2.81	(2.37)	1.17	(0.86)	3.29	(1.55)
Fe		0.2159 ns	3.73	(1.58)	10.62	(12.00)	2.90	(2.37)	9.42	(5.80)
K		0.4804 ns	50.53	(26.16)	150.45	(133.69)	417.79	(603.38)	1201.04	(2064.60)
Li		0.4803 ns	0.25	(0.23)	43.53	(59.48)	0.23	(0.17)	0.73	(0.62)
Mg		0.0472	11.46	(0.78)	18.58	(7.32)	42.72	(27.89)	66.48	(85.79)
Mn		0.1747 ns	0.40	(0.06)	0.50	(0.36)	0.32	(0.35)	1.07	(0.99)
Na		0.4314 ns	185.70	(197.06)	830.26	(1013.50)	250.95	(296.16)	524.78	(194.88)
P		0.8662 ns	39.04	(4.95)	95.31	(96.09)	58.36	(39.73)	148.32	(206.54)
Pb	0.7450 ns		1.03	(0.23)	0.76	(0.53)	1.08	(0.29)	0.99	(0.79)
Zn		0.1084 ns	6.24	(3.68)	17.99	(14.33)	3.44	(4.45)	9.12	(4.49)

Mean of replicated samples and standard deviation (S.D.). For each variable, the significance of differences between areas was tested by one-way ANOVA or Kruskal-Wallis rank sum test (Kr.-Wal.), for normally distributed and not normally distributed data respectively; ns, non-significant; different superscript letters indicate significant differences between areas tested by LSD *post-hoc* test after ANOVA.

**Figure 2:**
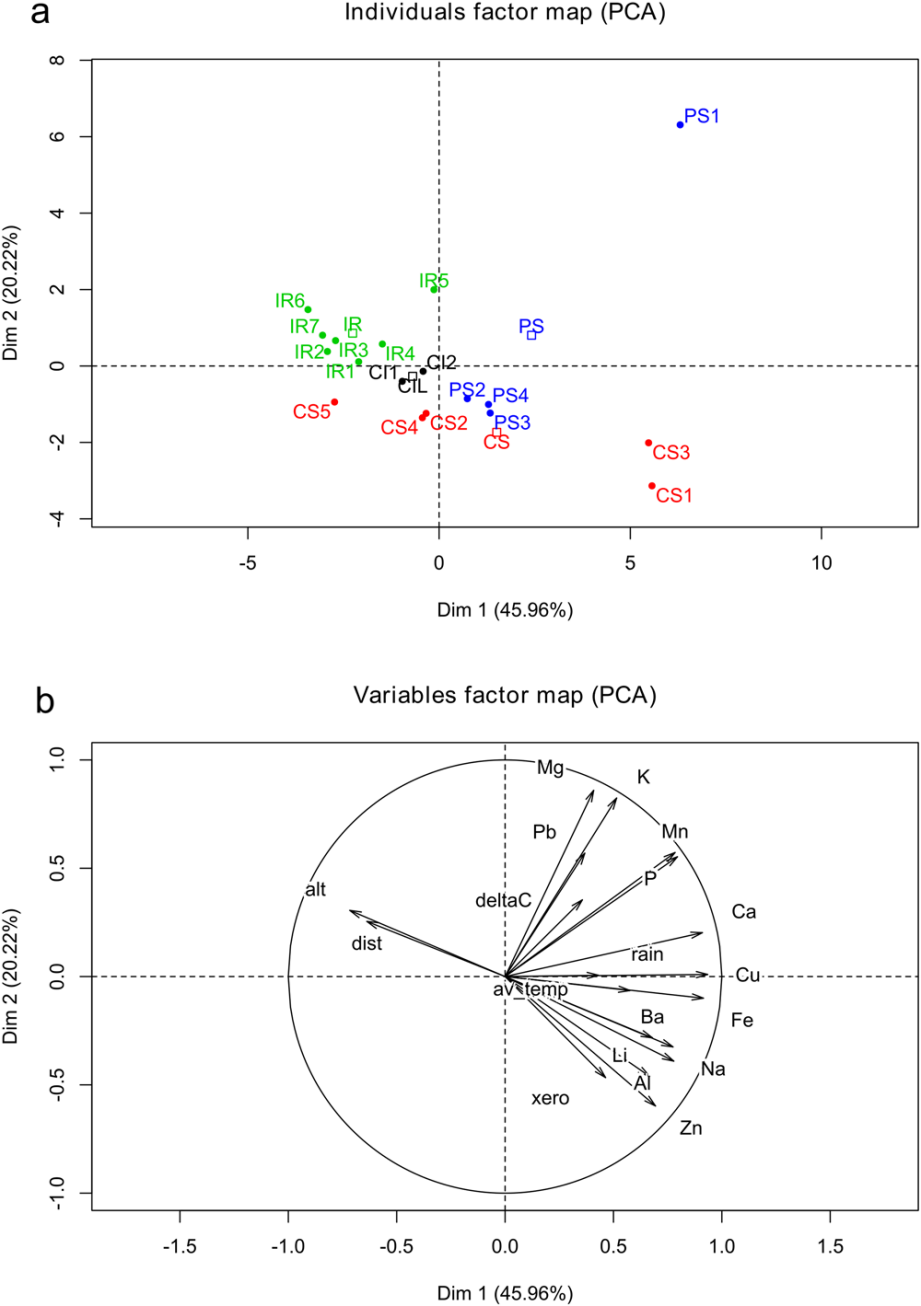
Principal Component Analysis on dataset of environmental variables of cultivation site and chemical features of oil samples from four production areas of the olive cultivar *Ogliarola campana* in Campania region: (**a**) score plot of oil samples. Abbreviations of production areas: CI, Cilento; CS, Colline Salernitane; IR, Irpinia; PS, Penisola Sorrentina. (**b**) Loading plot of variables. Alt., altitude; delta C, δ^13^C; dist., sea distance; av_temp., average temperature; xero, xerothermic index; elements are indicated by their chemical symbol.

The environmental features of the production areas and the corresponding chemical features of EVOOs were included in a multivariate analysis by PCA (Fig. 2). The first two dimensions of PCA express 64.8% of the total variance, a relatively high and significant value that explains a relevant part of the data variability. The first PCA dimension (44.59% of total variance) clearly opposes the samples PS1, CS1, and CS3 to some IR samples (IR2, IR3, IR6, and IR7). The second PCA dimension (20.23% of total variance) separates three groups: the first represented only by the sample PS1, the second one including the IR samples and the third one including an admixture of CI, PS and CS samples. The Ascending Hierarchical Classification reveals four significant clusters: cluster l, enclosing all IR samples, except IR4; cluster 2, including only CS5; cluster 3, enclosing CS1 and CS3; cluster 4, including only PS1. Globally, the PCA results highlight the distinctiveness of the IR samples, which are closely clustered and well separated from all the others. The three CI samples, finally, are closely grouped but admixed with CS and PS samples.

### Historical analysis

Here we present the main results of the historical analysis and a full version with detailed results is provided as Supplementary information 1. The etymology of the word “*Ogliarola*” is not mentioned in the specialized literature^25,26^. However, its origin could be explained by five hypotheses: 1) palatalization of the original term “*druppa oleariola*”, meaning “little olive fruit for oil production”; 2) the Latin *olea*, which is the ancient name of the olive tree^31^, whilst *oleum* is the oil, *-ara* indicates a collective and *-ola* is a diminutive; thus, the overall etymological meaning of *Ogliarola* could be “little plantation of olive”; 3) the dialectal word *ugghialoru*, which indicates a “little pot usually made of polished or tinned terracotta, used to contain oil for daily consume”^32^; 4) a woman working in the olive mill (*oliaria* or *olearia*) is nicknamed *oliarola* in an ancient document^33^; 5) *oliarolo* was the name given to the area around Bari in the XV century Current Era (CE), with reference to a district specialized in olive oil production, with relevant olive cultivation and commerce.

The toponym search identified a total of 151 terms with semantic correspondence to *Ogliarola* and olive cultivation: 49 terms in Irpinia area, 48 terms in Colline Salernitane, 30 terms in Cilento, and 24 terms in Penisola Sorrentina (Table 2). The highest number of results was retrieved in the category agricultural contracts (46), while much less elements were found in the categories historical toponyms and *Hospitia et hospitalia*, with only 10 and five occurrences, respectively. The four productions areas were heterogeneous in the partitioning of the six categories. In Irpinia, Penisola Sorrentina, and Colline Salernitane, the richest results were in agriculture contracts and historical places, whereas in Cilento the most represented terms were in present toponyms and *stationes*. It is interesting to notice that historical toponyms corresponding to the keyword *Ogliarola* were found only in Irpinia (Ogliara, close to Serino, Avellino) and Colline Salernitane (Ogliara, close to Salerno).

**Table 2:** Historical analysis on four production areas of the olive cultivar *Ogliarola campana* in Campania region.

	Productions areas			
	Cilento	Colline Salernitane	Irpinia	Penisola Sorrentina
**Variables**				
Agricultural contracts	2	20	15	9
Historical toponyms	0	2	8	0
Historical places	2	11	11	7
Present toponyms	14	11	5	2
*Stationes*	11	3	9	4
*Hospitia et hospitalia*	1	1	1	2

Results of search for keywords related to *Ogliarola* and olive cultivation.

**Figure 3:**
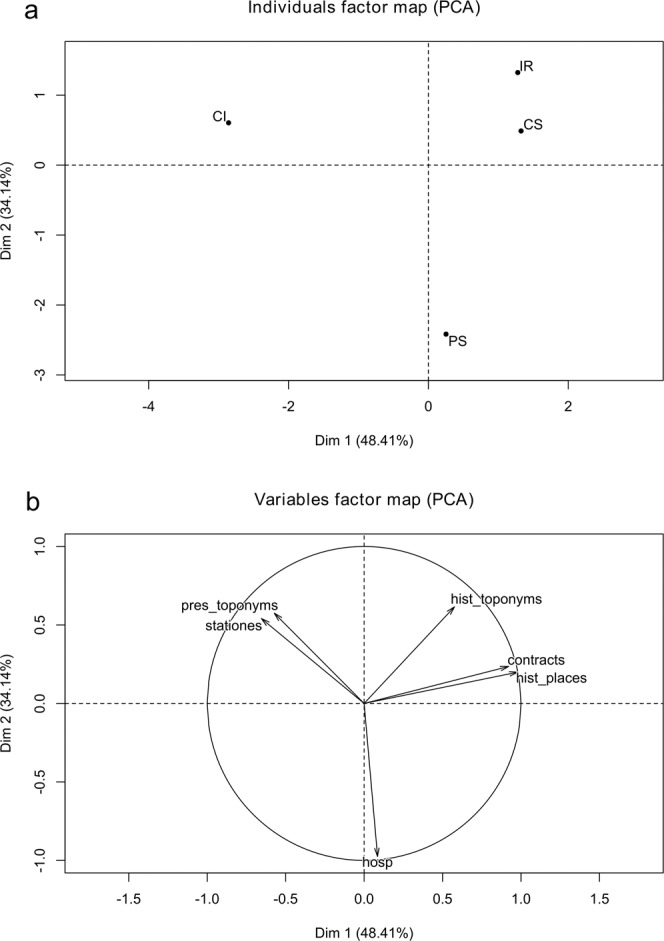
Principal Component Analysis on dataset of cultural-historical data from ancient documentation of four production areas of the olive cultivar *Ogliarola campana* in Campania region: (**a**) score plot of production areas; Abbreviations: CI, Cilento; CS, Colline Salernitane; IR, Irpinia; PS, Penisola Sorrentina. (b) Loading plot of variables. Abbreviations: hist_., historical; pres_, present; hosp., *hospitia et hospitalia*.

The multivariate analysis by PCA summarized the historical information and provided a global description of its diversity (Fig. 3). The first two dimensions of PCA explain a cumulative 82.6% of the total variance. Therefore, they largely describe the data variability. The score plot of samples (Fig. 3a) displays a small cluster (IR-CS) enclosing Irpinia and Colline Salernitane, whereas Penisola Sorrentina (PS) and Cilento (CI) are well separated. The first PCA dimension expresses 48.41% of variance and markedly separates Cilento from the other PDO areas. Particularly, the variable that significantly correlates with the first dimension is the number of historical places (R^2^ 0.98). The IR-CS cluster is characterized by a high number of historical places, whereas CI presents a low number of historical places. The second PCA dimension well separates PS from the other samples. The variable that significantly (R^2^ 0.97) correlates with the second dimension is the number of *hospitia et hospitalia*. Penisola Sorrentina is well separated, having twice the *hospitia et hospitalia* than the other areas. However, the number of *hospitia et hospitalia* was very limited (1 or 2) for all the 4 territories. Therefore, the observed variation pattern of this variable is not robust against small random fluctuations. Overall, PCA of historical variables clearly separates Cilento and Penisola Sorrentina, whereas Irpinia and Colline Salernitane were clustered by similar features.

### Terroir score of the Ogliarola PDO areas

The weight of the anthropic variables and the syntropy value of the physical variables of each PDO area are presented in Table 3 and the respective plots of *terroir* score are displayed in Fig. 1. Irpinia PDO shows the highest *terroir* score, and Penisola Sorrentina the lowest one (Fig. 1). The sensitivity analysis (Figs. S6 and S7) indicated that, as conservative approach, differences in *terroir score* greater than five are not likely to be caused by chance or random fluctuations. The shape of the radar plots indicates very distinct biocultural profiles of the four *Ogliarola* PDO areas. The plot highlights the biocultural complexity of Irpinia, which presents a balanced profile, with positive scores in all the variables, whereas the other PDO areas have one or more variables with null score. Penisola Sorrentina is mainly characterized by *Hospitia et hospitalia* and poor in other variables. Cilento is characterized by present toponyms, *Stationes*, and δ^13^C. Colline Salernitane is characterized by historical places and present toponyms.

**Table 3:** Evaluation score of four production areas of the olive cultivar *Ogliarola campana* in Campania region.

		Production Areas							
		Cilento		Colline Salernitane		Irpinia		Penisola Sorrentina	
**Anthropic variables**	Label	*V*_*k*_^*(n)*^	*W*_*k*_^*(n)*^	*V*_*k*_^*(n)*^	*W*_*k*_^*(n)*^	*V*_*k*_^*(n*)^	*W*_*k*_^*(n)*^	*V*_*k*_^*(n)*^	*W*_*k*_^*(n)*^
Agricultural contracts	ACON	2	0.100	20	1.000	15	0.750	9	0.450
Historical toponyms	HTOP	0	0.000	2	0.250	8	1.000	0	0.000
Historical places	HPLC	2	0.182	11	1.000	11	1.000	7	0.636
Present toponyms	PTOP	14	1.000	11	0.786	5	0.357	2	0.143
Stationes	STTN	11	1.000	3	0.273	9	0.818	4	0.364
Hospitia et hospitalia	HOHO	1	0.500	1	0.500	1	0.500	2	1.000
**Physical variables**		**Syntropy value**							
δ^13^C syntropy	D13C	1.000		0.039		0.311		0.500	
Elemental syntropy	ELEM	0.577		0.204		0.186		0.430	
**Score**		**49**		**42**		**53**		**30**	

For the first six anthropic variables, the left column reports the number of observations *V*_*k*_^*(n)*^ and the right column reports the weight *W*_*k*_^*(n)*^. For the last two physical variables, the syntropy value is reported in table. The second column shows the short label of each variable, as reported in the radar plots of Fig. 1.

## Discussion

We characterized the biocultural identity of the *Ogliarola campana* mono-varietal EVOO in four production areas of Campania region. As a first step, we conducted an analytical study of geographic and bioclimatic data, carbon isotope composition (δ^13^C) and elemental composition of EVOOs from the four cultivation areas. We show here how this investigation strategy allows characterizing the geography of the olive landscape at a regional scale. The analysis of trace elements was previously used in the geographic characterization of olive oils^14,34,35^. The geological profiles and lithostratigraphic units from ISPRA geological maps (Table 4) provided the information to interpret the element concentration in the *Ogliarola* oil samples. The presence of ignimbrite in the volcanic lithostratigraphy of Campania region characterizes some geological formations of the study area (tufo grigio campano – TGC and Vesuvian and Campi Flegrei system – VEF_1_) and could explain some balanced associations of elements (Mg, Pb and K; P and Mn; Ca and Fe)^36^. The presence of Ca is associated with gypsum in soils of Irpinia^37^, Penisola Sorrentina (Arenarie Del Deserto – ADD), and Cilento, characterized by calcareous marls (San Mauro formation - MAU). The red clays, commonly found in Colline Salernitane (Supersintema Eboli – CE), indicate the presence of Fe (pyroclastic elements) in the alluvial lithofacies. It is noteworthy that some elements detected in olive oils could derive from agronomic practices, such as K and P from fertilization and Cu from fungicides. Therefore, their concentration in the oil could be not exclusively linked to the soil composition. In our study, Cu concentration presented the highest variance and strongly contributed to the first dimension of PCA. K concentration was also highly discriminating on the second dimension of PCA. The effect of fertilizers and chemicals on the natural concentration of some elements is not quantified but some effect on oil composition can be expected. It is likely that a multivariate analysis is robust enough against contaminations in some elements, but caution is needed when interpreting elemental data for traceability of market products.

**Table 4:** Geological formations of four production areas of the olive cultivar *Ogliarola campana* in Campania region.

Production area	Geological formations
Irpinia	Molasse di Anzano – Membro di Flumeri, Subsintema della Montagna di Carife, Flysch Galestrino, Flysch Rosso, Formazione della Baronia
Penisola Sorrentina	Sintema Vesuviano-Flegreo, Arenarie del Deserto, Tufo Grigio Campano
Colline Salernitane	Supersintema Eboli
Cilento	Formazione di San Mauro

Despite the overall qualitative relationship between the element composition of oil and the geological nature of cultivation areas, significant differences between the four *Ogliarola* production areas were found only in carbon isotope composition (δ^13^C) and Ba of oil. Globally, the trace elements analysed were not able to discriminate the origin of mono-varietal EVOO samples of this case study. It is likely that, at the small geographic scale of Campania region, the clines of soil and climatic conditions are not variable enough to significantly affect the olive oil composition. Only Ba discriminated the four production areas, being higher in the samples from Penisola Sorrentina and Colline Salernitane (cluster PS-CS) than in Cilento and Irpinia samples. The values observed are in agreement with those reported in other studies on olive oils^9,14^. The most common minerals containing Ba are well known, barite (BaSO_4_), hollandite (Ba_2_Mn_8_O_16_) and witherite (BaCO_3_), but few information is available about the distribution of Ba in soils^38^. The geographical pattern of Ba found in our oil samples is in agreement with the natural abundance of Ba minerals in the soils of Penisola Sorrentina and Colline Salernitane (Table 3), rich in volcanic and clay formations^39^. This element is a good marker of soil composition as it does not occur as ionic form in natural waters and its concentration in natural aquatic systems depends on the mineral matrix^39,40^.Our study confirms Ba content as an interesting element to distinguish olive oils at a small geographic scale and makes it a discriminating indicator of EVOO geographic origin in Campania region. However, a mapping of trace element concentration in agricultural soils is needed to interpret the elemental composition of olive oil as marker of geographic origin. The other analytical feature that discriminated the production areas is δ^13^C, a marker related to plant water-use efficiency. It is an indicator of plant response to the local environment and is particularly sensitive to drought effects^12,41^. Actually, the significant variation in δ^13^C is consistent with the geographic pattern of bioclimatic variables among the four production areas. Thus stable isotopes of carbon are useful to characterize the ecological profile of olive oils at a regional scale.

A clearer characterization and discrimination of the *Ogliarola* production areas was obtained by multivariate PCA applied on the whole set of environmental and chemical variables. PCA shows a compact clustering of Irpinia oil samples, thus indicating their homogeneous features and their separation from the other areas. The oil samples from Penisola Sorrentina and Colline Salernitane showed a heterogeneous profile and overlapped in the PCA score plot, with some isolated samples being characterized by extreme PCA clustering. The oil samples from Cilento shared a similar multivariate profile, but were not separated from Penisola Sorrentina and Colline Salernitane. The clustering pattern of PCA reflects the geologic and climatic distribution of the production areas. The more distinct cluster of Irpinia, characterized by specific mountain conditions, is opposite to the coastal clusters of Penisola Sorrentina and Colline Salernitane. The Cilento group, characterized by high altitude and low distance from the sea, is in intermediate geographic position and closely associated to Colline Salernitane in PCA (Fig. 2a).

As a second step of our study, the biocultural postulate^27^, which integrates science and humanities aspects, was applied to discriminate the four *Ogliarola* production areas by analysis of cultural and historical elements. We started with the etymological analysis of the word “*Ogliarola*”, which was not previously reported in literature^25,26^. All the five hypotheses about *Ogliarola* etymology equally suggest the existence of a well-established olive oil supply chain in the medieval Campania region. The etymology analysis substantiates the biocultural approach and provides semantic tools for the search of historical terms by appropriate keywords.

The search for old agricultural contracts focused on the medieval period (IX to XIV century CE). The relatively high number of medieval contracts associated to olive groves found in our study indicates an ancient tradition of olive cultivation in Campania region (Table 2). This information is relevant for the cultural geography of the present olive cultivation. From the IX to the XIV century CE, this region was troubled and subjected to different dominations. Thus, the notary deeds about properties, including olive orchards, represented a social warranty for the land owners^42^. The number of agricultural contracts was elevated in Irpinia and Colline Salernitane, the areas that currently produce most of *Ogliarola*. These areas also showed a high number of present toponyms and historical places quoted in the historical sources, testifying the relevance of olive oil production in the past.

The search for toponyms also provided elements related to *Ogliarola campana*, suggesting that this cultivar is a vector of biocultural heritage for olive cultivation. The numerous toponyms found in this small area constitute a semantic network related to *Ogliarola* and olive tree^24^. We found only two toponyms that match the keyword “*Ogliarola*”, Civita di Ogliara in Irpinia and Ogliara in Colline Salernitane. Civita di Ogliara is the most important toponym corresponding to *Ogliarola* and identifies an archaeological site located in Serino municipality, rich in historical traces and sources^43^. It was built in 839 CE as defensive structure and also represented an outpost along a commercial route in the Longobardian Age. The history of local routes is relevant to understand the past evolution and the present geography of olive cultivation and trade. Contrary to the rich documentation about Civita di Ogliara, the toponym Ogliara found in Colline Salernitane matches just few historical references. The origin of this settlement is Etruscan^44^, but the tradition of olive cultivation is relatively recent and is not addressed to any medieval origin^45^. The interpretation of old regional maps helped reconstructing the origin of the two toponyms Ogliara found in our survey^46,47^. We can hypothesize different origins for the two toponyms: Civita di Ogliara, in Irpinia, would be related to a well-established olive oil supply chain in the past, particularly addressed to trade. In contrast, the toponym Ogliara in Colline Salernitane would not represent a trace of an ancient olive oil value chain, but it is likely related to a recent land use change in favour of olive orchards.

The last categories of historical elements included in the biocultural analysis were the sites or localities identified in the past as *stationes*, and *hospitia et hospitalia. Stationes* were the posts that supported the connectivity among cities and villages, where travellers found rest and services^48^. *Hospitia et hospitalia* were structures dedicated to the care of poor and infirm people^49^. The historical data related to these “social” variables provide information about the flux of people, including pilgrims, armies, merchants and travellers. The main routes and itineraries identified by *stationes*, and *hospitia et hospitalia* were also associated to the trade of olives and olive oil. The similarity in the number of *Hospitia et hospitalia* indicates similar social relevance of these structures among the four production areas. Indeed, the high number of *stationes* in Cilento could be explained by the wildness of this area in the Middle Ages and is in agreement with the prevalence of modern, rather than ancient toponyms and places (Table 2).

The complex information from historical sources has been summarized by the multivariate PCA, which clustered Irpinia and Colline Salernitane, according to their similar features, whereas Penisola Sorrentina and Cilento were well separated. The PCA also highlighted the agricultural contracts as a very informative variable, which well describes the intangible heritage and cultural diversity in the area of production. The analysis of toponyms and historical information suggests that Irpinia and other areas in Campania differ according to the succession of religious and political powers, which left different cultural footprints and affected the history and geography of olive cultivation. The multivariate analysis provides an overall statistical description of qualitative data of historical, geographic and cultural nature. However, such analysis does not fully depict the complexity of a *terroir* according to the biocultural concept. Few previous studies attempted to quantify the biocultural diversity and biocultural interactions in urban and rural landscapes. Winter and co-workers^50^ applied the concept of quantum co-evolution unit to quantify the biocultural elements of social-ecological systems in Hawaiian biocultural landscapes. Fukamachi and co-workers^51^ investigated the biocultural diversity of small scale landscapes in Satoyama (Japan) by multivariate analysis of vegetation data, land use patterns, traditional management techniques and cultural features. With a similar intention, the third step of our study aimed at obtaining a science-based whole measure of the biocultural elements that substantiate a novel concept of *terroir* in EVOO landscapes. For this, we set up a new *terroir score* (Fig. 1), which integrates analytical, cultural and historical variables in a single number and a clear plot indicating how a production area emerges as a typical production spot of *Ogliarola* EVOO. The *terroir score* indicates that Irpinia area has a high biocultural complexity and a balanced diversity of physical and anthropic variables. On the opposite, Penisola Sorrentina shows the lowest *terroir score*, due to an unbalanced richness of bio-cultural variables.

To obtain the new *terroir score*, we developed an original mathematical method that integrates heterogeneous variables and provides a numerical index and a plot showing pictorially the biocultural complexity of the *Ogliarola* production areas: the bigger and the less skewed the plot area, the better the score. The area of the plot is scaled according to the maximum value, therefore the method can be flexibly applied to any variables, quantitative or qualitative, and independently from the unit of measure and distribution of data. A caveat, however is the variable order in the radar plot, which affects the evaluation of the score. In order to overcome this difficulty, the score is calculated as the maximum of all the possible variable permutations, albeit in the graphical presentation all the plots have the same variable order for the sake of clarity. The radar plots allow an easy interpretation of the biocultural complexity of *Ogliarola* PDO. An irregular shape of the radar plot makes graphical representation tricky and suggests a non-robust profile, that could be the result of random values, especially when the number or raw occurrences or sample size is low. The sensitivity analysis indicated that the differences in *terroir score* between the PDO areas are not likely to be caused by chance. Further biocultural analyses based on our model would benefit of a solid statistical design that cannot be attained with the present *Ogliarola* dataset. This *terroir score* is an attempt to summarize the heterogeneous information from oil analytical features and historical-cultural elements and provides a new description of the regional olive landscape and a new concept of *terroir*. In fact, the general formula of biocultural diversity^29^ is based on variables, as language and religions, observed on large geographic areas but is not suitable to small regional areas, as our case study. By assessing the diversity of physical and anthropic data in small areas, the new *terroir score* overwhelms the global meaning of the general biocultural diversity and provides a novel index, which integrates a high number of biocultural variables.

In conclusion, the original *terroir score* proposed here is useful to characterize the identity and geography of local olive oil production areas by combining historical and anthropic elements with environmental and chemical features. The novel biocultural approach of the *terroir score* is a promising methodology to substantiate the *terroir* concept with science-based elements. The principle and the method could be applied to other cases and this novel concept of *terroir* well fits small scale areas, which are rich of cultural and historical information associated to traditional productions, as EVOOs. The results and the new method represent meaningful implements for the PDO concept and regulation. By this approach, the PDO significance would be based on both scientific and biocultural elements. In this way, a PDO would reflect not only the material value of a commodity but also its intangible values, providing resilience to local productions and rural societies, in agreement with UNESCO Florence Declaration^28^.

## Methods

### Sample collection

Eighteen samples of mono-varietal *Ogliarola campana* EVOO were directly collected in Campania region (Southern Italy), among olive production areas ascribed to four regional PDOs (Fig. 1): Irpinia (IR, 7 samples), Colline Salernitane (CS, 4 samples), Penisola Sorrentina (PS, 5 samples) and Cilento (CI, 2 samples). The unbalanced experimental design was constrained by local and uncontrolled factors, such as the uneven number of farms with presence of the cultivar and the varying willingness of producers to be recruited in the study. The *Ogliarola campana* cultivar (also referred to as *Ogliarola*) is also known as *Uogliarola* and *Minucciola* in the Sorrento peninsula (Penisola Sorrentina) and as *Ogliara* in Cilento. The samples of EVOO were individually collected from olive mills during 2013 harvest and each sample was representative of some farms located in the surrounding area. The mono-varietal identity of *Ogliarola campana* was verified in the orchards where the olives came from, by morphological identification of olive trees, with the support of local agronomists. Three random samples of EVOO were collected from each olive mill and delivered to the analytical laboratories in bottles of dark glass, then stored at room temperature for some weeks, until analysis. The geographic coordinates of each EVOO sample were referred to the olive mill position.

### Determination of carbon isotope and elemental compositions

The analysis of stable carbon isotopes was performed with a CF-IRMS (Isoprime GV, Elementar UK Ltd, Cheadle, UK) coupled with an elemental analyser (NA1500; Carlo Erba, Milan, Italy). In details, sub-samples of about 500 μg of oil were quantitatively combusted in the elemental analyser. The resulting CO_2_ was admitted through helium continuous flow to the isotope ratio mass spectrometer. The determination of the isotope ratios (R = ^13^C/^12^C) of both samples and internal standards allowed the calculation of the carbon isotope composition (δ^13^C) values of the sample, anchored to the reference scale of IAEA standard VPDB. Both gaseous (RM 8562, RM 8563, RM 8564) and solid IAEA standards (NBS-22 fuel oil and IAEA-CH6 Sucrose) were used for pristine determinations of the internal standards on the VPDB scale. The isotopic determinations were then expressed as δ notation, which was calculated as the relative deviation of the isotope ratio of a sample from that of the international standard, using the expression: δ = (R_s_– R_std_)/R_std_, where R_s_ is the isotope ratio of the sample and R_std_ is the isotope ratio of the international standard. The standard deviation of replicate measurements was ± 0.1‰. The obtained δ^13^C values, as usual, were multiplied by 1000 and shown in “per mil” units (‰).

The analysis of elemental composition was performed with a ICP-OES (optical emission spectrometry) iCAP 7200 instrument (Thermo Scientific Inc., Waltham, MA, USA), equipped with an ASX-520 auto sampler (Cetac Technologies Inc., Omaha, NE, USA). The samples were prepared and analysed according to the method of Camin *et al*.^9,10^. Briefly, 15 g of oil were extracted with 10 ml of extracting solution, which was prepared with 6,7% H_2_O_2_, 1% HNO_3_ and 0,2% HCl, in ultrapure water. The elemental analysis was carried out using radial and axial acquisition of the elements. The trace elements evaluated in the analysis were among the most frequently detected in olive oil, according to Beltran *et al*.^14^. The limit of detection (LOD) of each element was calculated as three times the standard deviation of the signal recorded in ten replicates of the blank samples. The LOD ranged from 0.06 µg/kg (Ba) to 45.65 µg/kg (Na). The relative standard deviation (RSD%) of the analytical method was calculated on five replicated extractions and analyses of an oil sample. The RSD% ranged from 2% (Ca) to 36% (Al).

### Climatic and geographic data

The database of the regional agricultural service (Regione Campania, Assessorato Agricoltura, Italy) was used to obtain climatic data from meteorological stations representative of the four production areas: Mirabella Eclano (IR), Forio d’Ischia (PS), Battipaglia (CS) and Stella Cilento (CI). The xerothermic index X_i_^28,29^ of each site was calculated using the formula:

$${{\rm{X}}}_{i}=\sum (2{{\rm{T}}}_{{\rm{med}}}-{\rm{P}}){\rm{if}}2{{\rm{T}}}_{{\rm{med}}} > {\rm{P}}\,{\rm{or}}\,{{\rm{X}}}_{i}=2{\rm{if}}2{{\rm{T}}}_{{\rm{med}}}\le {\rm{P}}$$

where, T_med_ is the monthly mean temperature and P is the monthly precipitation in mm.

The local climatic data of the sampling areas were interpolated from Worldclim datasets (www.worldclim.org). Other GIS layers include the administrative boundaries from Regione Campania (https://sit2.regione.campania.it/content/ctr), the digital elevation model and the aerial photographs from Ministero dell’Ambiente (http://www.pcn.minambiente.it). The Qgis GIS software^52^ was used to retrieve latitude, longitude, altitude and distance from the sea of the sampling sites. The geological characterization of the soils was derived from Carta Geologica d’Italia 1:50000, sheets: 433, 466, 467 and 503, issued by ISPRA^53^. The setting of a final biocultural map, in accordance with the previous evaluation and validation of the variables utilized, was performed in R^54^, DataGraph form Visualtools (https://www.visualdatatools.com/DataGraph/) and finally Qgis^52^.

### Cultural and historical analysis

We set up a method to analyse toponymy. Some anthropic and cultural elements related to the olive tree were taken as biocultural indicators, assuming that they are preserved in a semantic frame. This method combines information from medieval sources and from toponymy, in order to characterize the contemporary biocultural landscapes. Starting from the etymology of the word *Ogliarola*, we carried out a bibliographic search in the agricultural contracts of the Middle Ages within the current production areas of *Ogliarola* in Campania. In some cases, we also examined the relevant terms occurring in the history of certain areas (e.g., *Montis Corvini*) that do not correspond to the current localization of olive groves. This historical period is testified by an abundant written documentation about agricultural landscapes. The keywords and word roots utilized to investigate the toponyms of vegetation (phyto-toponymy)^25^ were related to *Ogliarola* (*Ogli-*) and to the olive plant (*oliv*-, *olib*-, *olev*-, etc.) (see the etymology by Rhizopolou^55^). We also investigated the terms related to practices of olive cultivation and oil production. For instance, the toponyms *Zapino* and *Torchiara* are referred to the orchard cultivation practices and to the oil extraction method, respectively.

The *Codices*, i.e. the documental manuscripts collected in a book of agricultural and other kind of contracts, were also examined: *Codex Diplomaticus Verginianus* (CDV, referring to Irpinia), *Codex Diplomaticus Amalfitanus* (CDA, referring to Penisola Sorrentina), *Codex Diplomaticus Cavensis* (CDC, referring to Colline Salernitane and Cilento) and Codice Solothurn (CoS, referring to Colline Salernitane and Cilento). In the case of CDV, the comprehension of historical sources was supported by the librarians of the *Biblioteca del Monumento Nazionale di Montevergine* (Mercogliano, Italy). Several original texts were found in the volume of Filangieri di Candida^56^. On-line resources provided by ALIM (*Archivio Latinità Italiana nel Medioevo*) project were utilized for the reading and interpretation of CDC.

The historical search was limited to the sources dating back to the medieval period, from IX to XIV century Current Era (CE). A cultural-historical database was set up for each current production area of *Ogliarola* and allowed the count of the following elements, also included in the evaluation of the *terroir score*:

- agricultural contracts,
- historical toponyms,
- historical places with past presence of olive orchards,
- present toponyms,
- medieval cities or stations (*stationes*),
- medieval structures for care of poor people (*hospitia et hospitalia*).

We considered the present toponyms when referred to practices of cultivation specifically associated to olive orchards (e.g., *Torchiara* and *Sanza*, close to Salerno) but we ignored those related to generic agricultural practices (e.g., *Pastena*, from the Latin verb *pastenare*, which means to plough). Furthermore, we took into account all the toponyms tightly linked to the cultivar *Ogliarola*.

### Statistical analysis and Score evaluation

Quantitative analytical variables (elemental composition, climatic data, stable isotope composition) were tested for normality of data distribution by Shapiro-Wilk normality test using R^54^. Data distribution was considered significantly deviating from normality at p-value <0.01. The normally distributed variables were further analysed by ANOVA and Fisher’s Least Significant Difference (LSD) *post-hoc* test using R. The variables with not normal distribution were analysed by the non-parametric Kruskal-Wallis rank sum test using R. Principal Component Analysis (PCA) was performed on quantitative data and on the historical dataset using R and FactoMineR package^57^. Samples and variables with missing data were excluded *a priori* from the PCA.

In order to obtain a single index representing both anthropic data and analytical measurements on *Ogliarola* EVOOs, we propose an original score evaluation method, based on the relative counting for the anthropic observations and on the Shannon’s information for the physical variables. A detailed description of this method, with literature citations, is provided in the Supplementary information 2, which also includes a Python script of the algorithm. Here we present the principle and a simplified description of the main steps. Briefly, we reduced the bulk of data to eight variables only, scaled within the 0…1 range (the greater the better). The values were plotted along the arms of a radar plot, then a synthetic score was evaluated, according to the area spanned by the plot. We applied the score evaluation procedure to the anthropic and physical variables:

Anthropic variables: the six variables (number of observations) are scaled to 0…1, according to the maximum value of the variable in all the PDOs, dividing the number of observations of a given variable by the maximum one. Table 3 contains, for each PDO, the number of observations and the scaled value. For ease of reading of the radar plots, each variable has been assigned an abbreviation label, defined in Table 3.

Physical variables: Each PDO has a distribution of the measurements over a certain number of *Ogliarola* EVOO samples. The information content of such distribution is evaluated in terms of Shannon’s information or *syntropy*, a “negative version” of the entropy: a higher typicality of the values is associated to a higher syntropy^58,59^. We selected the δ^13^C (treated as a single variable) and the elemental composition (averaged over all the elements) as relevant physical variables. We chose these variables because the δ^13^C is correlated to plant physiology and is an indicator of the climatic, geomorphologic and agricultural conditions. The presence of trace elements is also related to the plant cultivation environment and to the management practices. Given the large number of the elemental measurements, we summed up these variables, averaging the respective syntropies.

The final score, named *terroir score*, was evaluated according to the area of the radar plot (Fig. 1). Each arm of the plot represents the value of a variable. The plot area is scaled with respect to the maximal regular polygon (an octagon in this case), so that if all the variables relative to a PDO have a value of one, the scaled area measures exactly one. On the other hand, if the radar plot area is shrunk (all or nearly all the variables with zero-value) the area is close to zero. The score is finished by a logarithmic transformation to compensate saturation of the highest values and expressed in percent of the maximum allowed area. Due to the experimental design of our case study, without replicated samples in each PDO area, it was not possible to estimate a confidence interval of the *terroir score*. In order to assess the score reliability, we conducted a sensitivity analysis with an ensemble of Gaussian perturbations to a set of simulated measurements (Supplementary information 2). The graphical output of the radar plots was done using DataGraph form Visualtools (https://www.visualdatatools.com/DataGraph/). The *terroir score* and the associated plot characterize the biocultural profile and the typicality of a given production area.

## Supplementary information

Supplementary information.
