# Supplementary material for: Advances in biocultural geography of olive tree (Olea europaea L.) landscapes by merging biological and historical assays
Source: Sci Rep. 2020 May 6;10:7673. doi: 10.1038/s41598-020-64063-8 (PMC7203204; doi:10.1038/s41598-020-64063-8)
Supplement: Supplementary file 1 — Supplementary information. [file 41598_2020_64063_MOESM1_ESM.pdf]

Supplementary information

Advances in biocultural geography of olive tree (*Olea europaea* L.) landscapes by merging biological and historical assays

Giuseppe Russo[a*], Isacco Beritognolo[a], Marina Bufacchi[b], Vitale Stanzione[b], Andrea Pisanelli[a], Marco Ciolfi[a], Marco Lauteri[a] and Stephen B. Brush[c]

[a] Institute of Research on Terrestrial Ecosystems (IRET), National Research Council of Italy (CNR), Viale Marconi 2 – 05010 Porano (TR), Italy

[b] Institute for Agricultural and Forest Systems in the Mediterranean (ISAFoM), National Research Council of Italy (CNR), Via della Madonna Alta 126 – 06128 Perugia, Italy

[c] Department of Human Ecology, University of California, Davis, One Shields Avenue, Davis, CA 95616, USA

* corresponding author, e-mail <giuseppe.russo@iret.cnr.it>

Supplementary information 1

Analysis of historical elements and toponyms

Introduction

In this document, we present a part of the study that addresses the biocultural diversity of extra-virgin olive oil (EVOO) production areas of the Italian region Campania. This region, owing to its geographic and historical complexity, represents an ideal study area to develop a novel paradigmatic interpretation of the EVOO biocultural diversity, which considers the oil analytical features and the cultural and historical elements of the territory (Agnoletti, 2006). The olive cultivar *Ogliarola campana* is well represented in four EVOO Protected Designations of Origin (PDO) of Campania region: Irpinia-Colline dell'Ufita, Penisola Sorrentina, Colline Salernitane and Cilento (Di Vaio, 2012). Campania region has a structured EVOO value chain and the historical importance of olive oil production is well documented since the Roman Age, as reported by Pliny the Helder in the *Naturalis Historia*. Later, the Christian monasticism drove the transformation of uncultivated lands, reprising the olive cultivation after the instability due to the invasions and the wars of the Early Middle Ages (Dalena, 2010). In the medieval southern Italy, the strong influence of monasticism on the agriculture sector is testified by ancient sources and toponymy. Campania region is rich of toponyms related to agriculture, which form a complex "semantic network" revealed by their etymology (Aversano, 2001). The local toponyms with archaic origin reveal a background related to the late Latin or to the vernacular of the Early Middle Ages (Pellegrini, 1990; Pellegrini 2008), when the monasticism promoted the development of olive oil production in Campania. This rich geographic and historical information could aid in dissecting and interpreting the present biocultural diversity of EVOO landscape. The concept of biocultural diversity bases on the assumption of an inextricable link between biological (environment, fauna and flora) and cultural (history, religion, ethnics and language) diversity (Maffi, 2007). The UNESCO Florence Declaration (2014) recognizes the social importance of the biocultural diversity and recommends actions at regional level to implement certification and product labelling, and promote the competitiveness of local productions and rural landscape. However, due to their limited extension, the local olive production systems cannot be interpreted according to the standard requirements of the biocultural diversity concept, which includes variables of humanity richness (e.g., languages, ethnicity, religion, technology) at a large

geographic scale (Loh and Harmon, 2005). On the contrary, the toponymy can characterize production areas at a small scale and reveal the diversity of the local intangible heritage in terms of ancient practices of local land use. The bio-environmental and cultural complexity of Campania region appears adequate to overcome the intrinsic limitations of biocultural studies at a small geographical scale (Roßmann, 2013) and to adopt an analytical-classificatory-semantic study of toponyms (Siniscalchi, 2008).

Methods

We set up a method to analyse toponymy and historical sources. Some olive-related elements were taken as biocultural indicators, assuming that they are preserved in a semantic frame. This method combines information from medieval sources and from toponymy, in order to characterize the contemporary biocultural landscape. Starting from the etymology of the word *Ogliarola*, we carried out a bibliographic search in the agricultural contracts of the Middle Ages within the current production areas of *Ogliarola* in Campania. However, in some cases, we examined the relevant terms occurring in the history of certain areas (e.g., *Montis Corvini*) that do not correspond to the current localization of olive groves. This historical period is testified by an abundant written documentation, which highlights a diversity of agricultural landscapes. The keywords and word roots utilized to investigate the toponyms of vegetation (phyto-toponymy) (Pellegrini, 1990) were related to *Ogliarola* (*Ogli-*) and to the olive plant (*oliv-*, *olib-*, *olev-*, etc.) (see the etymology by Rhizopolou, 2007). We also investigated the terms linked to practices of olive cultivation and oil production. For instance, the toponyms *Zapino* and *Torchiara* are referred to the orchard cultivation practices and to the oil extraction method, respectively.

The *Codices*, i.e. the documental manuscripts collected in a book of agricultural and other kind of contracts, were also examined: *Codex Diplomaticus Verginianus* (CDV, referring to Irpinia), *Codex Diplomaticus Amalfitanus* (CDA, referring to Penisola Sorrentina), *Codex Diplomaticus Cavensis* (CDC, referring to Colline Salernitane and Cilento) and Codice Solothurn (CoS, referring to Colline Salernitane and Cilento). In the case of CDV, the comprehension of historical sources was supported by the librarians of the *Biblioteca del Monumento Nazionale di Montevergine* (Mercogliano, Italy). Several original texts were found in the volume of Filangieri di Candida (CDA, 1917). On-line resources provided by ALIM (*Archivio Latinità Italiana nel Medioevo*) project were utilized for the reading and interpretation of CDC.

The historical search was limited to the sources dating back to the medieval period, from IX to XIV century Current Era (CE). A cultural-historical database was set up for each current production area of *Ogliarola* and allowed the count of the following elements, also included in the evaluation of the *terroir* score:

- agriculture contracts,
- historical toponyms,
- historical places with past presence of olive orchards,
- present toponyms,
- medieval cities or stations (*stationes*),
- medieval structures for care of poor people (*hospitia et hospitalia*).

We considered the present toponyms when referred to practices of cultivation specifically associated to olive orchards (e.g., *Torchiara* and *Sanza*, close to Salerno) but we ignored those related to generic agricultural practices (e.g., *Pastena*, from the Latin verb *pastenare*, which means to plough). Furthermore, we took into account all the toponyms tightly linked to the cultivar *Ogliarola*, e.g., *Civita di Ogliara* (Irpinia) and *Ogliara* (North of Salerno).

Results and Discussions

We characterized the biocultural identity of the *Ogliarola campana* mono-varietal EVOO in four production areas of Campania region, according to the the biocultural postulate (Maffi, 2007), which integrates science and humanities aspects of a whole. Therefore, we analysed the cultural and historical elements from the *Ogliarola* production zones.

We started with the etymological analysis of the word "*Ogliarola*", which was not previously reported in the specialized literature (Pellegrini, 1990; Pellegrini 2008). Its origin could be explained by five hypotheses: 1) palatalization of the original term "*druppa oleariola*", meaning "little olive fruit for oil production"; 2) the Latin *olea*, which is the ancient name of the olive tree (Gledhill, 2008), whilst *oleum* is the oil, *-ara* indicates a collective and *-ola* is a diminutive; thus, the overall etymological meaning of *Ogliarola* could be "little plantation of olive"; 3) the dialectal word *ugghialoru*, which indicates a "little pot usually composed by polished or tinned terracotta, used to contain oil for daily

consume" (Cortelazzo and Marcato, 2005); 4) a woman working in the olive mill (*oliaria* or *olearia*) is nicknamed *oliarola* in an ancient document (Di Muro, 2005); 5) *oliarolo* was the name given to the area around Bari in the XV century, with reference to a district specialized in olive oil production, with relevant olive cultivation and commerce. All the five hypotheses about *Ogliarola* etymology equally suggest the existence of a well-established olive oil supply chain in the medieval Campania region. The etymology analysis substantiates the biocultural approach and provides semantic tools for the search of historical terms by appropriate keywords.

The historical and toponymy analysis focused on the medieval period (IX to XIV century CE). The toponym search identified a total number of 151 terms with semantic correspondence to *Ogliarola* and olive cultivation: 49 terms in Irpinia zone, 48 terms in Colline Salernitane, 30 terms in Cilento, and 24 terms in Penisola Sorrentina (Tab. S1). The highest number of results was retrieved in the category agricultural contracts (46), while much less elements were found in the categories historical toponyms and *Hospitia et hospitalia*, with only 10 and five occurrences, respectively. The four productions zones were heterogeneous in the partitioning of the six categories. In Irpinia, Penisola Sorrentina, and Colline Salernitane, the richest results were in agriculture contracts and historical places, whereas in Cilento the most represented terms were in present toponyms and *stationes*. The relatively high number of agricultural contracts associated to olive groves of the Early Middle Age indicates an ancient and solid tradition of olive cultivation in Campania region. This information is relevant for the cultural geography of the present olive cultivation. The period from the IX to the XIV century was highly dynamic in this region, which was subjected to different dominations. In such a troubled historical frame, the development of notary deeds about properties, including olive orchards, represented a social warranty for the land owners (Di Muro, 2013). The number of agricultural contracts found in the medieval *Codex* was particularly elevated in Irpinia and Colline Salernitane, the areas that currently produce most of *Ogliarola*. These areas also presented a high number of present toponyms and historical places quoted in the historical sources, testifying the relevance of olive oil production in the past.

The search for toponyms also provided remarkable elements related to *Ogliarola campana*, suggesting that this cultivar is a vector of heritage for the biocultural geography of olive cultivation. The relatively high number of toponyms found in this small area constitutes a semantic network related to *Ogliarola* and olive tree (Aversano, 2001). We found only two toponyms matching the keyword "*Ogliarola*", Civita di Ogliara in Irpinia and Ogliara in Colline Salernitane. Civita di Ogliara is the most

important toponym corresponding to *Ogliarola* found within the semantic network. The toponym Civita di Ogliara identifies an archaeological site located in Serino (lat. 40.83°N; long. 14.90°E; elev. 615 m a.s.l.). Here we report the key facts about the history of this site. Civita di Ogliara was built in 839 CE as defensive structure, during the war that contrasted the Longobardian Principality of Count Siconolfo and Count Radelchi (Baranowski, 2002). This political occurrence strongly affected the biocultural evolution of Campania region in the next centuries. Civita di Ogliara also represented an outpost along a commercial route in the Longobardian Age and the history of the local routes is relevant to understand the past evolution and the present geography of agricultural land use, including olive cultivation. Contrary to the rich documentation about Civita di Ogliara, the toponym Ogliara found in Colline Salernitane matches just few historical references. Although the origin of this settlement is Etruscan (Greco Pontrandolfo, 1980), the tradition of olive cultivation in this area is relatively recent and is not addressed to any medieval origin (De Crescenzo, 1949). The interpretation of old regional maps helped reconstructing the history of the two toponyms Ogliara found in our survey. Giustiniani (1805) reported the term Ogliara in Irpinia only. Nevertheless, olive plantations were not present in the ancient site of Civita di Ogliara, but rather oak, beech and chestnut woods characterized the area, as they currently do (Bellabona, 1642). Therefore, we can hypothesize different origins for the two toponyms Ogliara. In Irpinia, Civita di Ogliara would be related to a well-established olive oil supply chain in the past, particularly addressed to marketing and trade. In contrast, the toponym Ogliara found in Colline Salernitane would not represent a trace of an ancient olive oil value chain but it is likely related to a recent land use change in favour of olive orchards.

The Middle Age documents referring to the old civilizations and their socio-political dynamics are a relevant source to understand the history and geography of olive cultivation. In Campania, coastal olive groves are widely spread and related to the history of diverse civilizations: Byzantines, *Ecclesia* and Normans (Dalena, 2010). In the transition from Roman to Middle Age, olive oil represented a symbol of the Roman's cultural heritage. As such, it was preserved in its fundamental nutritional and commercial values by the new Longobardian dominators in Southern Italy. Furthermore, they extended the use of olive oil to the liturgical and religious ceremonies, in addition to its traditional role in food preparation (Montanari, 2007). In the last part of the historical period investigated (XIII century CE), the political power shifted from the Longobardian to the Christian Church *Ecclesia salernitana* (the synod of the *salernitanae diocesae*). As a consequence, the ancient route linking the neighbouring Principalities of

Benevento (inner mountain area) and Salerno (coastal area) decreased in importance, with a relocation of the route towards Northwest, to cover other areas of interest (Di Muro, 2013). This change was coincident with the development of new olive cultivations by the Benedectine monks of Cava de' Tirreni (Dalena, 2010), the abbey that provided the *Codex Diplomaticus Cavensis* (CDC).

The analysis of historical elements included cities and localities that in the past were known as *Stationes* and *hospitia et hospitalia.* The *stationes,* evolution of the Roman *mansiones,* were the posts supporting the connectivity among cities and villages, where travellers found rest and services (Augenti, 2006). *Hospitia et hospitalia,* known as *xenodochia* in the Roman and Early Middle Ages, were structures dedicated to the care of poor and infirm people (*pauperes*) (Dalena, 2003). The historical data related to these "social" variables can provide useful information about the flux of people, including pilgrims, armies, merchants and travellers. The routes (*itinera*) were well-established since the Roman Age, often indicated by maps (i.e. the *Tabula Peutingeriana*) and chronicles. The similarity in the number of *Hospitia et hospitalia* indicates similar social relevance of these structures among the four production zones of this study. Indeed, the high number of *stationes* in Cilento could be explained by the wildness of that area in the Middle Ages. This is in agreement with the prevalence of modern, rather than ancient toponyms and places, found in the same area (Tab. S1).

The multivariate analysis by PCA summarized the historical information and provided a global description of its diversity (Fig. S1). The first two dimensions of PCA explain a cumulative 82.6% of the total variance. Therefore, they largely describe the data variability. The score plot of samples (Fig. S1a) displays a small cluster (IR-CS) enclosing Irpinia and Colline Salernitane, whereas Penisola Sorrentina (PS) and Cilento (CI) are well separated. The first PCA dimension expresses 48.41% of variance and markedly separates Cilento from the other PDO districts. Particularly, the variable that significantly correlates with the first dimension is the number of historical places (R^2 0.98). The IR-CS cluster is characterized by a high number of historical places, whereas CI presents a low number of historical places. The second PCA dimension well separates PS from the other samples. The variable that significant (R^2 0.97) correlates with the second dimension is the number of *hospitia et hospitalia.* Penisola Sorrentina is well separated, having twice the *hospitia et hospitalia* than the other zones. However, the number of *hospitia et hospitalia* was very limited (1 or 2) for all the 4 territories. Therefore, the observed variation pattern of of this variable is not robust against small random fluctuations. Overall, the multivariate PCA method provides an overall statistical description of qualitative data of historical,

geographic and cultural nature, and clustered Irpinia and Colline Salernitane, according to their similar features, whereas Penisola Sorrentina and Cilento were well separated. The PCA also highlighted the agricultural contracts as a very informative variable, which well describes the intangible heritage in the area of production influenced by cultural diversity. The analysis of toponyms and historical information suggests that Irpinia and other areas in Campania differ according to the succession of religious and political powers, which left different cultural footprints and affected the history and geography of olive cultivation. In combination with physical and analytical features (elemental composition and carbon stable isotope ratio of EVOOs), the historical and geographical variables contribute to characterize the identity and geography of local olive oil production areas.

References

Agnoletti, M. Introduction: framing the issue - a trans-disciplinary reflection on cultural landscapes. in *The conservation of cultural landscapes* (ed. Agnoletti, M.) pp. xi-xix (2006).

Augenti, A. *Le città italiane tra la tarda antichità e l'alto Medioevo: atti del convegno (Ravenna, 26 - 28 febbraio 2004).* (All'Insegna del Giglio, 2006).

Aversano, V. *Ricerca e didattica in geografia: dalla teoria all'applicazione*. (Elea Press, 2001).

Baranowski, T. Polish-Italian research in Southern Italy (Capaccio Vecchia and Civita di Ogliara). *Archaeologia Polona* **40**, 73–82 (2002).

Cortelazzo, M. & Marcato, C. Dizionario etimologico dei dialetti italiani: DEDI. (UTET Libreria, 2005).

Bellabona, S. *Ragguagli della città di Avellino, per Camillo Cavallo*. (1642).

Dalena, P. *Dagli Itinera ai percorsi: viaggiare nel Mezzogiorno medievale*. (Adda, 2003).

Dalena, P. *Mezzogiorno rurale: olio, vino e cereali nel Medioevo*. (Adda, 2010).

De Crescenzo, G. & Vernieri, N. *Dizionario salernitano di storia e cultura*. (1960).

Di Muro, A. *La Piana del Sele in età normanno-sveva: società, territorio e insediamenti (ca. 1070-1262).* (Adda, 2005).

Di Muro, A. *Signori e contadini nel Mezzogiorno normanno: il Codice Solothurn (fine sec. XII).* (Mario Adda editore, 2013).

Di Vaio, C. *Il germoplasma dell'olivo in Campania. Descrizione delle principali cultivar ed effetto dell'ambiente*. (Ed. Regione Campania, 2012).

Filangieri Di Candida, R. *Codice diplomatico amalfitano*. (Stab. Tipografico Silvio Morano, Napoli, 1917).

Giustiniani, L. *Dizionario geografico-ragionato del Regno di Napoli*. (Presso V. Manfredi, 1805).

Gledhill, D. *The names of plants*. (Cambridge Univ. Press, 2008).

Greco Pontrandolfo, A. Un gruppo di tombe di un insediamento rurale del IV sec. a.C. da S. Angelo di Ogliara (Salerno). in *Annali del Seminario di Studi del Mondo Classico. Sezione di Archologia e Storia Antica.* **II**, 39–112 (1980).

Loh, J. & Harmon, D. A global index of biocultural diversity. *Ecological Indicators* **5**, 231–241 (2005).

Maffi, L. Biocultural Diversity and Sustainability. in *The SAGE Handbook of Environment and Society* 267–278 (SAGE Publications Ltd, 2007). doi:10.4135/9781848607873.n18

Montanari, M. Olio e vino, due indicatori culturali. in 1–47 (CISAM, 2007).

Pellegrini, G. B. Variazione del paesaggio attraverso lo studio della fitotoponomastica. in *L'ambiente vegetale nell'alto medioevo* 549–588 (1990).

Pellegrini, G. B. *Toponomastica italiana: 10.000 nomi di città, paesi, frazioni, regioni, contrade, monti spiegati nelle loro origine e storia*. (Ulrico Hoepli, 2008).

Rhizopoulou, S. Olea europaea L.: A Botanical Contribution to Culture. American-Eurasian J. Agric. & Environ. Sci. 2, 382–387 (2007).

Roßmann, A. New approaches for verifying the origin of food. in *New analytical approaches for verifying the origin of food* (ed. Brereton, P.) 41–57 (Woodhead Publishing Limited, 2013).

Siniscalchi, S. L'identità 'svelata': un esempio d'indagine geografico-storica sul Cilento attraverso la toponomastica (secc. XVII-XIX). Geotema 12, 78–87 (2008).

UNESCO-SCBD. Florence declaration on the links between biological and cultural Diversity. in (UNESCO-SCBD, 2014).

Table S1. Historical analysis on four production areas of the olive cultivar *Ogliarola campana* in Campania region. Results of search for keywords related to *Ogliarola* and olive cultivation.

	**Productions areas**			
	**Cilento**	**Colline Salernitane**	**Irpinia**	**Penisola Sorrentina**
**Variables**				
Agricultural contracts	2	20	15	9
Historical toponyms	0	2	8	0
Historical places	2	11	11	7
Present toponyms	14	11	5	2
Stationes	11	3	9	4
Hospitia et hospitalia	1	1	1	2

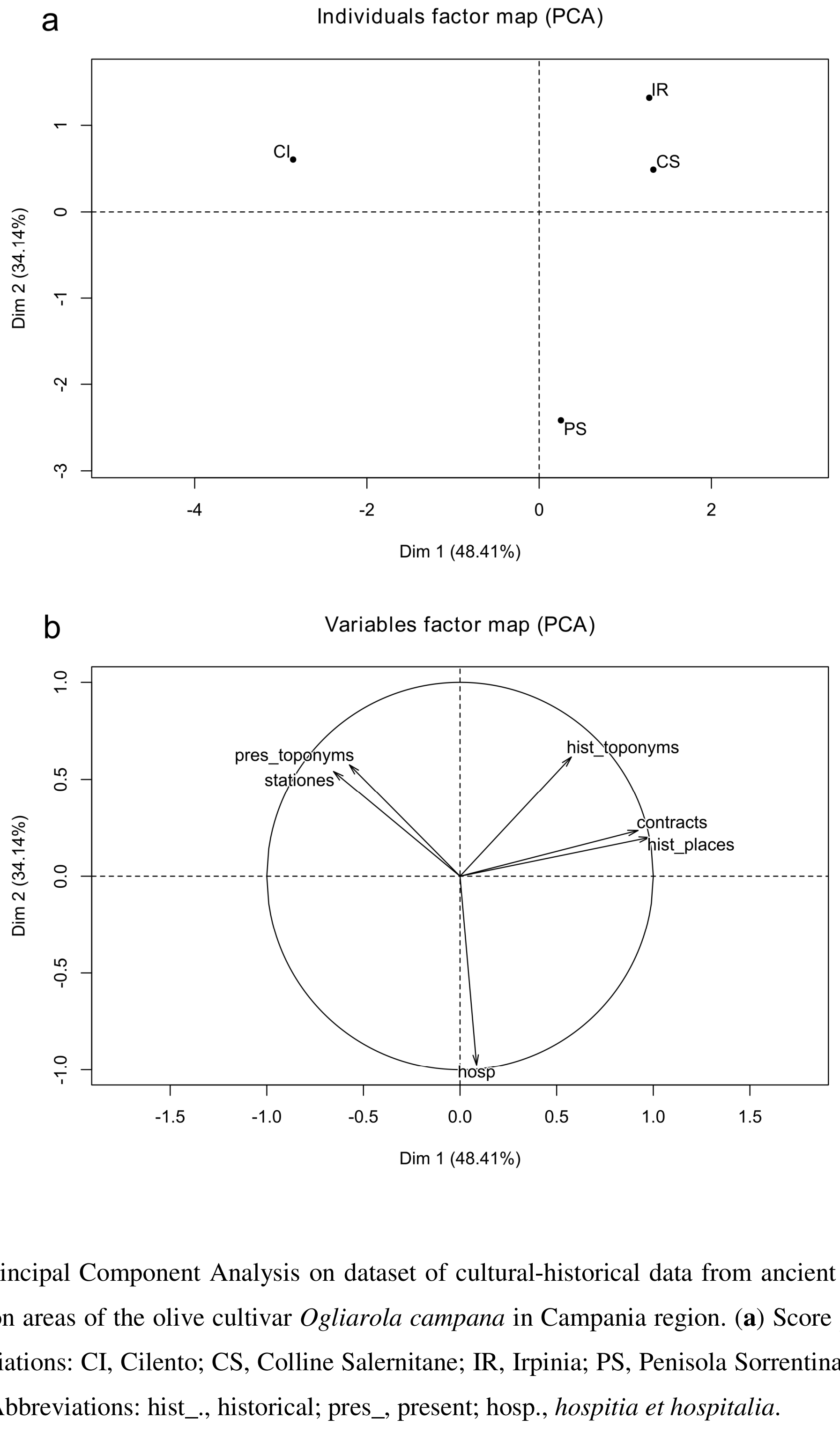

Figure S1. Principal Component Analysis on dataset of cultural-historical data from ancient documentation of four production areas of the olive cultivar *Ogliarola campana* in Campania region. (**a**) Score plot of production areas; Abbreviations: CI, Cilento; CS, Colline Salernitane; IR, Irpinia; PS, Penisola Sorrentina. (**b**) Loading plot of variables. Abbreviations: hist_., historical; pres_, present; hosp., *hospitia et hospitalia*.

Supplementary Information 2

Terroir Score

Procedure outline

In order to obtain a single index representing both cultural and historical variables (number of observations) and physical measurements (continuous random variables) linked to the otherwise blurry concept of *terroir*, we propose a score evaluation technique that measures the vocational character of a certain area with respect to other similar ones. Mixing qualitative and quantitative measurements always poses a number of issues, due to the heterogeneity of the statistical inputs.[1] This technique outputs a score between 0 (the least) and 100 (the most suited one); the starting point is a dataset of observations of anthropic and physical variables, each of which is transformed into a numerical value between 0 and 1. These values are then represented in a radar plot (also known as spider plot) and the inner area, rescaled in the 0…100 range, is taken as the *terroir score*. This algorithm can be used with any kind and number of variables from three on; as a rule of thumb, it is best suited for a number of variables between 5 and 20. The novelty of the proposed analytical methodology consists in the ability to integrate such different kinds of input variables, associating a score with the ease of reading of a conventional radar plot.

In short, the algorithm goes as follows; for each area to be evaluated:

- The anthropic variables are scaled to 0…1 dividing the area value by the maximum one. Zero means no observation at all and one represents the greatest number of observations.

[1] See T. P. Legendre and L. Legendre: *Numerical Ecology*, 3rd edition, Elsevier (2012), ch.5.

- The physical variables are used to derive the associated information content, or *syntropy*, a sort of reverse-entropy[2] that measures how typical the value is in a certain area with respect to the general distribution of the variable across all the areas. Also these values are scaled according to the maximum one. Any area has an associated distribution of values that can be associated to an entropy. Zero means a totally uninformative distribution of the variable, while one is associated to the highest level of typicality.
- All the transformed values are placed along the arms of a radar diagram, as in figure S2. The border of the plot is a regular polygon with a number of sides equal to the number of variables. The area of the polygon (shaded in fig. S2) is taken as the measure of the score. Zero means a totally shrunk area, while one is associated to the top value of all the variables[3]

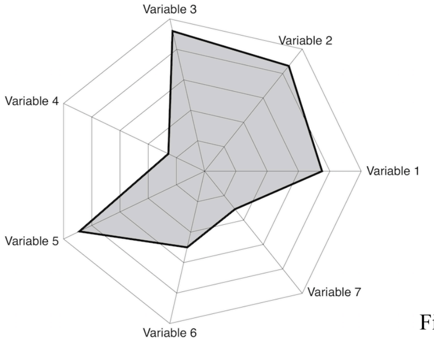

Figure S2

- The score is finished with a small logarithmic transformation of the area value,[4] compensating for some saturation of the highest values, followed by a multiplication by 100. The integer part is the *terroir score*. Of course, zero means no significant terroir-related anthropic-historical

[2] See the seminal paper C. E. Shannon: *A mathematical theory of communication*, Bell System Technical Journal, 27:379–423 (1948); for an updated introduction to the subject see R. Gray: *Entropy and Information Theory*, Springer (2011).
[3] The diagram perimeter gives some information, too: a small perimeter is associated with low variables values but a large perimeter can be associated to a large area or to an irregular shape as well, so that the perimeter alone is not enough, but it can be useful for a finer analysis of the *terroir score*. The area-to-perimeter ratio, for instance, is a measure of regularity of the shape, which can be interpreted as a robustness indicator of the index. Notice, however, that the maximum perimeter is not constrained to 1, using as a scaling factor the perimeter of the regular N-polygon.
[4] About 0.1 at most, on a 0…1 scale, thus promoting the lower-middle scores up to 10 points, keeping unchanged the lowest and highest scores.

observations and no typical values of the physical variables (i.e. maximum entropy), while the maximum score is only associated with a top placement of the area for every variable. The scores can be shown along the accompanying radar plots as in figure S3, which depicts an example of seven variables for three areas.

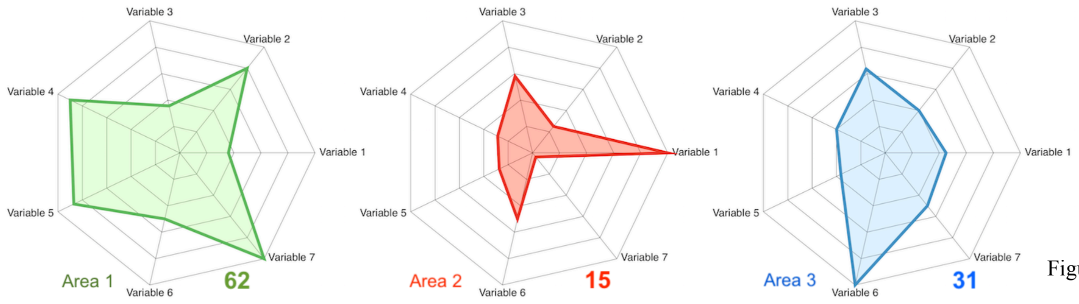

Figure S3

Terroir score algorithm

This rather technical section contains all the mathematical details of the algorithm. The procedure can be split into two steps: 1.Variables pre-processing and 2.Score evaluation. The accompanying source file radar.py contains a Python implementation of the score evaluation part.

Let's suppose that we have K geographical areas A_1 ... A_K. For any area A_k, $k = 1 \dots K$, we have M anthropic variables X_m whose values are X_m^k and N physical variables Y_n, whose distributions are Y_n^k, $m = 1 \dots M$, $n = 1 \dots N$. Note that the values of the anthropic variables are single integers, while the physical variables are distributions of real random variables: $Y_n^k = \{y_{n,j_n}^k\}$, $j_{kn} = 1 \dots J_{kn}$ where J_{kn} is the number of observations of the nth variable in the kth area. The total number of observations is thus $\hat{N} = \sum_k J_{kn}$. The difference between single observations and distributions is crucial, since we cannot define an entropy for a single observation.

1A. Pre-Treatment of the Anthropic Variables

For any anthropic variable, we define $x_m^k = \left[\max_k X_m^k\right]^{-1} X_m^k$ a trivial rescaling of the values based on the maximum found in all the areas for such variable. The new x_m^k remains 0 if the corresponding X_m^k was 0, its value is 1 if the original X_m^k was the maximum one, while the intermediate values are scaled accordingly. This simple rescaling of the values could be conducted in a much more sophisticated way, weighting the values according to the geographical extension or some anthropic variable, as the population count, the number of major cities or some other suitable one. If this is the case, instead of the $\{X_m^k\}$ one uses a new, derived set of variables $\{\tilde{X}_m^k\}$ defined as

$$\tilde{X}_m^k = \frac{1}{m(A_k)} X_m^k$$

where $m(A_k)$ is the measure of the weighting parameter.[5] The rescaling then proceeds as before.

1B. Pre-Treatment of the Physical Variables

This part of the procedure transforms a distribution in a variable in the 0…1 range. It is the most involved part of the procedure, that can be implemented in any statistical package or spreadsheet, according to the user's taste. For any variable Y_n, let's consider the set of all the observations of the variable in all the areas: this is a set of $\widehat{N}$single observations from which we derive a distribution according to the four main quantiles: 25%, 50% (the median) and 75%. This division into four classes is a matter of convenience, it has been chosen since the total number of observations $\widehat{N}$ can be quite

---

[5] For a comprehensive discussion of the Spatial methodologies see Noel A. C. Cressie: *Statistics for Spatial Data*, John Wiley & sons (1993) and Noel Cressie, Christopher K. Wikle: *Statistics for Spatio-temporal Data*, Wiley Series in Probability and Statitics, John Wiley & sons (2011).

small, so the use of finer classes leads to empty or singly-populated bins of the distribution histogram. For the sake of generality, said H the number of bins of the histogram, every distribution Y_n^k has an associated set of frequencies $Y_n^k = \{y_{n,j_n}^k\} \to \{f_{n,1}^k, f_{n,2}^k, \cdots f_{n,H}^k\}$ from these we evaluate the corresponding Shannon entropy for the nth variable in the kth area as[6]

$$S_n^k = -\sum_{h=1}^{H} f_{n,h}^k \log_2 f_{n,h}^k$$

The base of the logarithm is not really important, since the resulting values will eventually be rescaled. From the entropy we evaluate the *syntropy* or neg-entropy $\tilde{S}$ according to

$$\tilde{S}_n^k = \log_2 \sqrt{2\pi e\sigma^2} - S_n^k$$

Basically, $\tilde{S}$ is the difference in terms of information from a standard Gaussian distribution with the same mean and standard deviation σ. Here σ is assumed as the variance σ^2 of the Y_n distribution, over all the areas. [7] Again, the base of the logarithm is not important, as long as it is coherent with S. Now, we have transformed a distribution of observations Y_n^k into a single value $\tilde{S}_n^k$ that represents the information associated to Y_n^k. These $\tilde{S}_n^k$ are finally rescaled in order to constrain their upper range to one, thus obtaining, for each area, a set of N variables $s_n^k = \left[\max_k \tilde{S}_n^k\right]^{-1} \tilde{S}_n^k$ to be merged to the x_m^k.

---

[6] Note that the expression for the entropy is well-defined even if one has one or more empty histogram classes, posing $x \log x = 0$, as customary, since $\lim_{x \to 0^+} x \log x = 0$. Care should be taken, however, not trying to evaluate log 0 directly, especially in a spreadsheet. Some programming languages can handle infinities, but not indeterminate forms like $0 \cdot \infty$.
[7] See Gray 2011 (cit.)

2. SCORE EVALUATION

To each area A_k we associate a vector of $\boldsymbol{w}^k$ of $Q = M + N$ variables

$$\boldsymbol{w}^k = \left[w_1^k \cdots w_Q^k\right] = \left[x_1^k \cdots x_M^k, s_1^k \cdots s_N^k\right]$$

This "gluing" is algebraically consistent since all the quantities involved have homogenous values in the [0,1] closed interval. Once an order for the variables is chosen, the $\boldsymbol{w}$s are plotted along the arms of a radar diagram, as in figure S4.

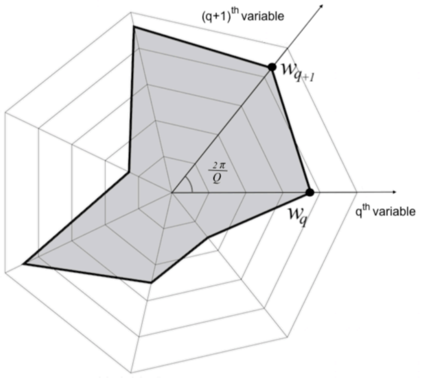

Figure S4

The radar area is evaluated as the sum of all the triangles which sides form a couple of contiguous variables, split apart by an angle of $\frac{2\pi}{Q}$. In general, for each $\boldsymbol{w}^k$ the corresponding radar area R_k is

$$R_k = \frac{1}{2\,P_Q}\ sin\frac{2\pi}{Q}\sum_{q=1}^{Q} w_q^k\ w_{(q+1)mod\ Q}^k$$

Where P_Q is the area of the regular polygon with N sides. The division by P_Q rescales the value of R_k within the [0,1] interval.[8] The previous formula is based on the summation of the areas of the triangles

[8] A Q-sides regular polygon corresponds to the maximum possible performance of all the variables. Anything less has a lower area, so a lower score.

that constitute the radar polygon; in fact, the product of two adjacent the sides lengths times the sine of the angle in between (i.e. their vector product) is twice the area of the triangle.

There is a subtle point, though. The R_k value thus defined depends on the order of the variables in the plot: a different arrangement of the variables gives a different estimate of R_k. To correct this biased estimate one must evaluate R_k considering all the possible arrangements of the variables, retaining the maximum,[9] although the radar plots are presented with the same variable order for clarity. The unbiased R_k is:

$$\tilde{R}_k = f_Q \max_{Perm(\boldsymbol{q})} \left\{ \sum_{q=1}^{Q} w_q^k \; w_{(q+1) mod\, Q}^k \right\}$$

where we have condensed the common factors into $f_Q = \frac{1}{2\, P_Q} \; sin \frac{2\pi}{Q}$, which is common to all the R_k. In fact, this f_Q is a function of the number of variables Q only.

Finally, the score for the A_k area is defined as follows:

$$\text{Score}(A_k) = \text{int}\left[100 \; \log_2\left(1 + \tilde{R}_k\right)\right]$$

The logarithm transforms the interval [0,1] in itself, with a slight amplification of the smaller values at the expense of those closer to 1. This small correction has been introduced to compensate for the accumulation of observations, promoting the areas with a smaller, but not zero, value of R. The factor 100 simply scales the score in the 1…100 range for ease of reading.

[9] It is also possible to take the average value of $\tilde{R}_k$ over all the permutations, or any other correction that takes into account all the possible arrangements of the variables, as long as it is applied coherently to all the areas.

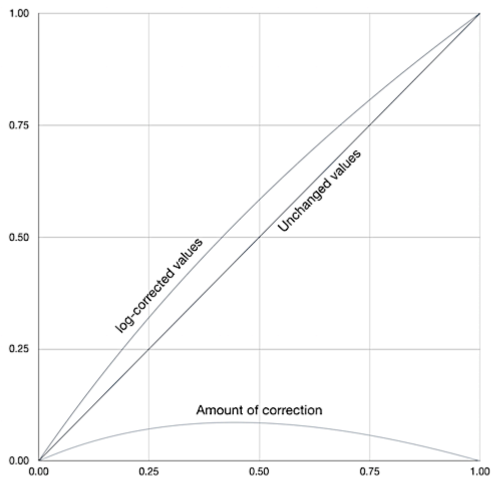

Figure S5

The logarithmic correction is shown in figure S5: the curve below, in particular, shows the amount of correction which is zero for an input value of zero and peaks a little before the median value, thus promoting the lower values more than the higher ones.

The Python script

We provide a collection of functions in Python3 for the evaluation of the terroir score. The accompanying code (radar.py) is a simple collection of functions for all the calculations described in score evaluation part of the previous section; as stated, the pre-treatment of the variables has to have been done beforehand. The Python functions are described in detail in the script itself. Other than the standard *math* library, the *itertools* library[10] is needed for the loop over the arrangements of the variables. The script allows the user to choose whether to use the logarithmic correction or not. It is also possible to compute the radar plot perimeter, as sketched in the procedure outline section.

The use of the script is straightforward: users should just edit the configuration and run the script.

[10] See https://docs.python.org/3/library/itertools.html.

```python
# ----------------------------------------------------------
#
#  RADAR INDEX EVALUATION - Python 3 script
#
#  areas are maximized according to all the permutations
#  of the axes for each zone. the area is normalized with
#  respect to the corresponding regular polygon
#  it is also possible to use the perimeter for index evaluation
#  setting the USE_AREA flag to false
#  use log2 values stretching if the values to promote smaller values
#
#  the number of variables for each zone con be different
#  (if it is meaningful / acceptable)
#
#  the index values are included in [0,1], where:
#    0 corresponds to all variables = 0
#    1 corresponds to all variables = 1 (maximum allowed input)
#  the score equals index * 100, rounded
#
#  the input variables values must be within [0,1]
#    [0...1] values are directly proportional to the score
#    a single variable <> 0 produces a zero index if areas are used
#
#  FLAGS:
#
#    SHOW_VARS = True: show, False do not show in output the variables values
#    USE_AREA = True: uses the area, False the perimeter to evaluate the radar size
#    LOG_STRETCH =  True: stretch with log2(x+1), False do not stretch the index value
#
#  USER VARIABLES:
#
#    every zone_k is ['label', value1, value2, ...]
#    zones is [zone_1, zone_2, zone_3, ...]
#
#
#  CREDITS & LICENSE:
#
#       Marco Ciolfi - may 2018 - CNR-IBAF - marco.ciolfi@ibaf.cnr.it
#
#       GNU-GPL 3 license
#
# ----------------------------------------------------------
#
#  FLAGS
#
SHOW_VARS = False
USE_AREA = True
LOG_STRETCH = True
#
#  VARIABLES
#
zone_1 = ['Z1', 0.2, 0.3, 1.0, 0.8, 0.0, 0.3]
zone_2 = ['Z2', 0.4, 0.3, 1.0, 0.8, 0.8, 0.7]
zone_3 = ['Z3', 0.4, 0.9, 1.0, 0.9, 0.5, 0.4]
zone_4 = ['Z4', 1.0, 1.0, 1.0, 1.0, 1.0, 1.0]
zone_5 = ['Z5', 0.0, 0.0, 0.0, 0.0, 0.0, 0.0]
zone_6 = ['Z6', 0.0, 0.0, 0.0, 0.0, 0.5, 1.0]
zone_7 = ['Z7', 0.0, 0.0, 0.0, 0.0, 0.7, 0.0]
#
zones = [zone_1, zone_2, zone_3, zone_4, zone_5, zone_6, zone_7]
#
# ----------------------------------------------------------

import math
import itertools

TWOPI = 2.0 * math.pi

#triangle third side (Carnot's Th)
def third_side(a, b, angle):
    """The third S of a triangle given SAS"""
    c = math.sqrt(a*a + b*b - 2.0 * a * b * math.cos(angle))
    return c

#n-sides regular polygon perimeter
```

```python
def perimeter_poly(n):
    """The perimeter of a regular n-gon inscribed in a unit circle"""
    perimeter = n * math.sqrt(2*(1 - math.cos(TWOPI / n)))
    return perimeter

#radar perimeter normalized to max regular polygon perimeter = 1
def perimeter_radar(sides):
    perimeter = 0.0
    num = len(sides)
    for k in range(0, num):
        a = sides[k]
        b = sides[(k+1) % num]
        perimeter += third_side(a, b, TWOPI/num)
    perimeter /= perimeter_poly(num)
    return perimeter

#triangle uarea = vector product / 2
def area_triangle(a, b, angle):
    """The area of a triangle, given SAS"""
    area = .5 * a * b * math.sin(angle)
    return area

#n-sides regular polygon area
def area_poly(n):
    """The area of a regular n-gon inscribed in a unit circle"""
    area = .5 * n * math.sin(TWOPI / n)
    return area

#radar area normalized to max regular polygon area = 1
def area_radar(sides):
    area = 0.0
    num = len(sides)
    for k in range(0, num):
        a = sides[k]
        b = sides[(k+1) % num]
        area += area_triangle(a, b, TWOPI/num)
    area /= area_poly(num)
    return area

#evaluates index, log-stretched if uselog is true
def eval_index(sides):
    index = 0.0
    for perm in list(itertools.permutations(sides)):
        index = max(index, (area_radar(perm)) if USE_AREA else perimeter_radar(perm))
    if(LOG_STRETCH): index = math.log2(index + 1.0)
    return index

#main loop
print('\nSTART\n-------------------\n')
for zone in zones:
    label = zone[0]
    sides = zone[1:len(zone)]
    index = eval_index(sides)
    score = round(100.0 * index)
    retstring = 'zone ' + label
    if SHOW_VARS: retstring += ' ' + str(sides)
    retstring += ' index = ' + str(index)
    retstring +=' score = ' + str(score)
    print(retstring)
print('\n-------------------\nDONE')

# -----------------------------------------------------------
```

3. Score Sensitivity Analysis

To assess the score reliability, we conducted a sensitivity analysis with an ensemble of gaussian perturbations to a set of simulated measurements. The ensemble is composed as follows:

- we consider up to 8 zones, $N_z = 2 \dots 8$
- we consider up to 8 variables, $N_v = 3 \dots 8$
- for each N_Z, N_v pair we consider 10 randomly generated measurements, with the variables uniformly distributed in the interval [0, 1]
- for each measurement we consider 1000 gaussian perturbations, 100 each for σ in the interval [0.1, 1.0] in 0.1 increments; μ is taken as the measurement value

the ensemble is thus composed of (8-2+1) × (8-3+1) × 10 × 10 × 100 = 420000 elements.

For each perturbation, we accumulate the total squared score residuals, from these we computed the average score fluctuation per zone, as a measure of the score reliability.

Our results show a general decreasing trend of the score fluctuation with increasing number of zones and variables, in particular (figures S6 and S7), for our case study $N_z = 4$ and $N_v = 8$: in this case the distribution of the score fluctuations is:

- first quartile: score fluctuation = 2
- median: score fluctuation = 2
- third quartile: score fluctuation = 4

the distribution is left-skewed, as it is apparent in figure S6.

These values suggest, as a conservative approach, that difference of scores of about 5 is significant within the sensitivity of the proposed score.

We include the python code of the ensemble sensitivity test.

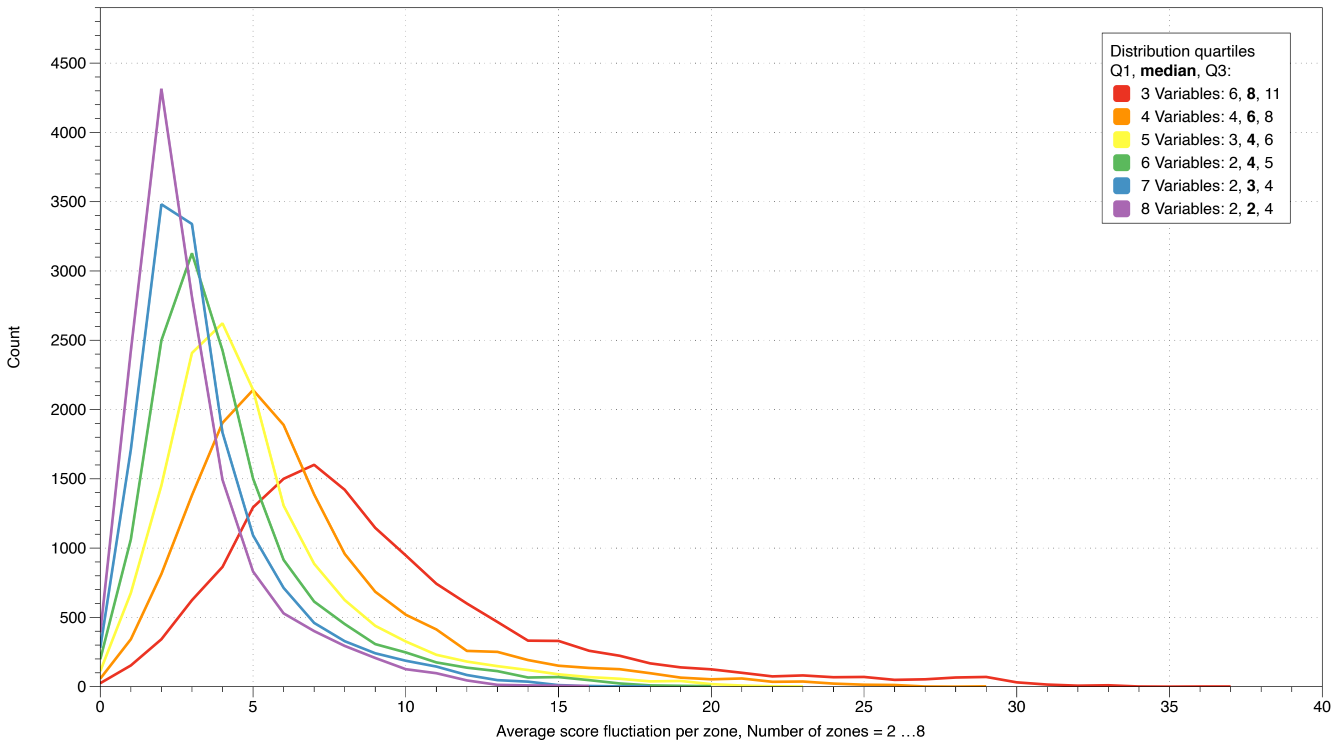

Figure S6

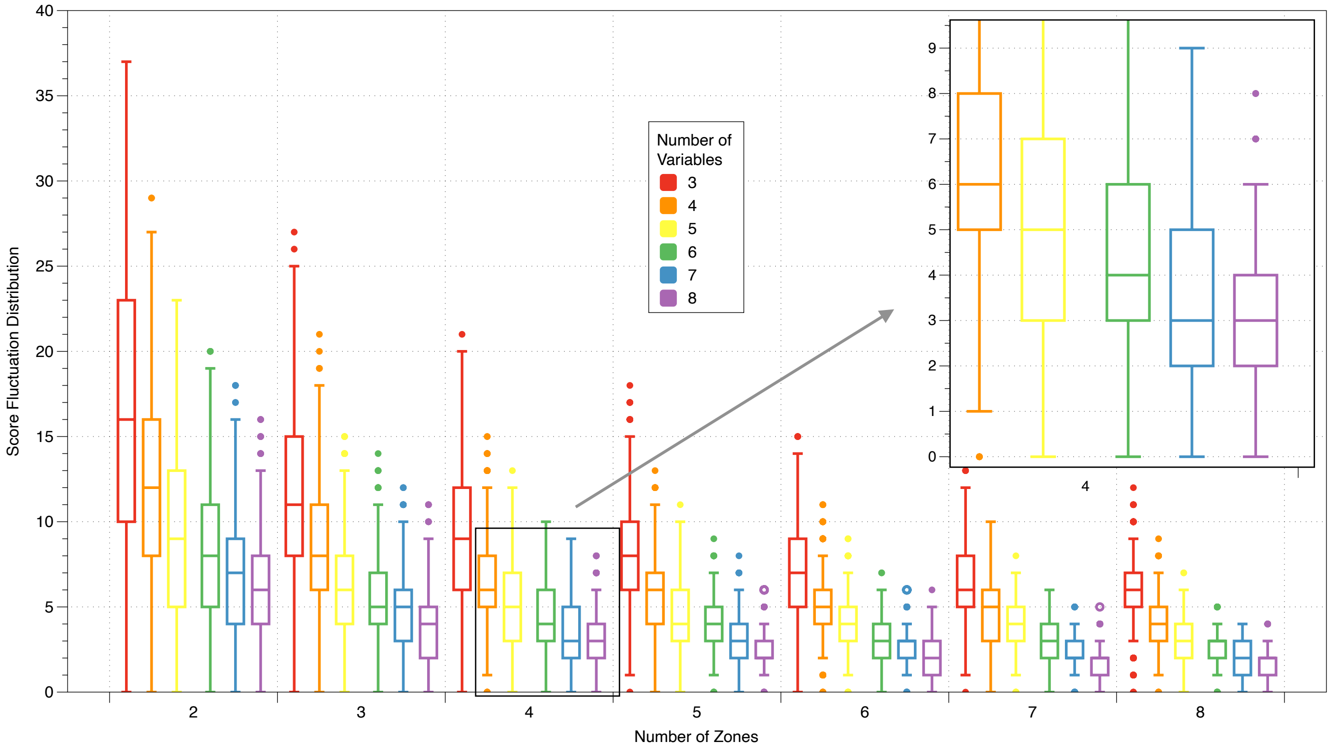

Figure S7

```
import math
import random
import itertools
from copy import copy
from datetime import datetime
from numpy import linspace
from math import sqrt
TWOPI = 2.0 * math.pi

ENS_NUM = 10
ENS_REPL = 100
OUT_FILE = './ensemble.txt'

POLY = 1.
SINE = 1.
SQRTN = 1.

def main():
    global SINE
    global POLY
    global SQRTN
    random.seed(datetime.now())
    with open(OUT_FILE, 'w') as out:
        out.write('NZ,NV,ENS,STDEV,SQRES,RESpZ,RESpV,RESpZV,ERR\n')
        for nv in range(3, 9):
            for nz in range(2, 9):
                SINE = math.sin(TWOPI / float(nv))
                POLY = 1. / (float(nv) * SINE)
                SQRTN = 1. / sqrt(float(nv))
                for sigma in linspace(.1, 1., 10): ensemble(nz, nv, sigma, out)

def ensemble(nz, nv, sigma, out):
    for ens in range(ENS_NUM):
        zones = []
        for k in range(nz):
            sides = []
            for v in range(nv): sides.append(random.uniform(0, 1))
            zones.append(sides)
        for v in range(nv):
            maxv = 0.
            for k in range(nz): maxv = max(maxv, zones[k][v])
            if maxv > 0:
                for k in range(nz): zones[k][v] /= maxv
        indices = []
        for sides in zones:
            indices.append(eval_index(sides))
        for repl in range(ENS_REPL):
            newzones = copy(zones)
            for z in range(nz):
                for v in range(nv):
                    newzones[z][v] = random.gauss(newzones[z][v], sigma)
                    newzones[z][v] = min(max(newzones[z][v], 0.), 1.)
            newindices = []
            for newsides in newzones:
                newindices.append(eval_index(newsides))
            sqres = 0.
            for k in range(nz):
```

```
                sqres += (indices[k] - newindices[k]) * (indices[k] -
                         newindices[k])
            res = sqrt(sqres)
            respz = res / float(nz)
            respv = res / float(nv)
            respzv = res / float(nz * nv)
            out.write('{},{},{},{},{},{},{},{},{}\n'.format(nz, nv, ens, c
                   clean(sigma), clean(sqres), clean(respz), clean(respv),
                   clean(respzv), int(100 * SQRTN * respz)))

def eval_index(sides):
    index = 0.0
    for perm in list(itertools.permutations(sides)):
        index = max(index, (area_radar(perm)))
    index = math.log2(index + 1.0)
    return index

def area_radar(sides):
    area = 0.0
    num = len(sides)
    for k in range(0, num):
        area += sides[k] * sides[(k+1) % num] * SINE
    area *= POLY
    return area

def clean(x: float):
    return round(x, 4)

if __name__ == '__main__': main()
```
